# Evaluation of serum level of C-reactive protein (CRP) and its correlation with fetal ultrasound parameters in the prediction of threatened miscarriage in the first trimester

**DOI:** 10.5339/qmj.2024.9

**Published:** 2024-02-21

**Authors:** Ahmed Mohamed Lotfy, Wael Soliman Taha, Muhamed Ahmed Abdelmoaty

**Affiliations:** 1 Department of Obstetrics and Gynecology, Senbillawen General Hospital, Egyptian Ministry of Health and Population, Egypt; 2 Department of Obstetrics and Gynecology, Faculty of Medicine, Al-Azhar University, Egypt Email: MuhamedAhmed.216@azhar.edu.eg

**Keywords:** reactive protein (CRP), fetal ultrasound parameters, threatened miscarriage, first trimester, Egypt

## Abstract

Background: Pregnancy loss occurring before 20 weeks gestation is referred to as miscarriage. Various clinical presentations of miscarriage include threatened, inevitable, incomplete, complete, septic, and missed miscarriage. Early-stage threatened miscarriage may manifest with symptoms such as abdominal discomfort and vaginal bleeding. Threatened miscarriage is clinically defined as the manifestation of positive fetal heart sounds in pregnancies occurring before the 20th week of gestation, concomitant with vaginal bleeding and a closed cervix.

Objectives: The primary aim of this study was to evaluate the association between serum C-reactive protein (CRP) levels and fetal ultrasound findings in the prediction of threatened miscarriage during the first trimester of pregnancy.

Methods: In this prospective case-control study, a total of 100 pregnant women at 7–13 weeks of gestation were enrolled. All participants initially presented with a singleton embryo displaying cardiac activity on ultrasound. The study cohort was divided into two groups: Group 1 consisted of 50 women with uncomplicated pregnancies, while Group 2 comprised 50 women experiencing symptoms indicative of threatened miscarriage.

Results: Notably, within Group 2, patients who eventually experienced miscarriage exhibited significantly elevated serum high-sensitivity CRP levels in comparison to those who maintained their pregnancies.

Conclusions: Threatened miscarriage cases demonstrated a substantial increase in serum high-sensitivity CRP levels compared to the control group. Furthermore, CRP levels exhibited a correlation with the risk of miscarriage, suggesting their potential utility in conjunction with ultrasound parameters for prognosticating threatened miscarriage during the first trimester.

## Introduction

Miscarriage, defined as the termination of pregnancy before 20 weeks of gestational age, encompasses various types, including threatened, inevitable, incomplete, complete, septic, and missed miscarriage.^1^ Among these, threatened miscarriage may manifest in early pregnancy with symptoms such as lower abdominal pain and/or vaginal bleeding. Approximately 25 percent of pregnant women experience some degree of vaginal bleeding during the first two trimesters, and roughly 50 percent of these cases progress to an actual miscarriage.^2^

According to two studies, 16.6% and 13.7% of threatening miscarriage instances experienced spontaneous miscarriages. The first was a case-control study in Turkey, while the second was a retrospective cohort study in a major healthcare facility in Nigeria.^3,4^

Numerous risk factors associated with miscarriage have been identified, including advanced maternal and paternal age, previous pregnancy losses, TORCH infections (Toxoplasmosis, Other (syphilis, varicella-zoster, parvovirus B19), Rubella, Cytomegalovirus (CMV), and Herpes infections), uncontrolled hyperglycemia, obesity, uncontrolled thyroid disease, significant stressors, the use of teratogenic medications, and the presence of a subchorionic hemorrhage. A comprehensive assessment of these risk factors in women presenting with threatened miscarriage can aid in determining prognosis.^5^

A threatened miscarriage raises the prospect of troubling outcomes, not only for the pregnancy itself but also for the developing fetus. Research indicates that women experiencing bleeding or other signs suggestive of impending miscarriage are statistically more prone to complications like premature labor, abnormal placenta placement, and pregnancy-related high blood pressure. Should the pregnancy advance to viability despite early alarms, the lingering risks still include diminished fetal growth and postnatal hospitalization in intensive care. A threatened loss, even if ultimately avoided, thus alerts care providers to heighten monitoring and support.^6^

Ultrasound examination has become the gold standard for monitoring early pregnancy development and complications. Several sonographic parameters, such as gestational sac diameter, yolk sac diameter, crown-rump length, and fetal heart rate, are pivotal for prognosticating early pregnancy outcomes. Serial ultrasound assessments enable close monitoring of embryo/fetal development and viability.^7^

C-reactive protein (CRP) is a plasma protein that rises in the acute phase in response to inflammation. CRP, an ancient protein found in all animal species, is important in innate immunity because it binds pathogens, activates complement, and stimulates phagocytosis. CRP measurement clinically shows the intensity of inflammation and tissue damage^8^

Recent evidence has also suggested a potential role for maternal serum high-sensitivity C-reactive protein (CRP) in various pregnancy-related applications. Elevated CRP levels have been linked to first-trimester pre-eclampsia screening,^9^ predicting long-term cardiovascular risks in women with late pregnancy hypertensive disorders^10^ and diagnosing early-onset neonatal infection associated with chorioamnionitis.^11^

This study addresses a significant gap in existing research by focusing on the correlation between C-reactive protein (CRP) levels and fetal ultrasound parameters in predicting threatened miscarriage during the first trimester. While previous studies have explored low-grade inflammation in miscarriage cases, our primary objective is to enhance predictive capacity by investigating the combined role of serologic and sonographic markers. This approach aims to provide a deeper understanding, potentially leading to improved prognostication of pregnancy outcomes.

## Patients and Methods

### Study design

This prospective case-control study was conducted from July 2022 to September 2023 at the Gynecological and Obstetric Department of Al-Hussein University Hospital. The study recruited 100 pregnant women within the gestational age range of 7–13 weeks, all exhibiting initial ultrasound evidence of a singleton embryo displaying detectable cardiac activity.

### Participant enrollment

Participants were meticulously screened and categorized into two distinct groups. Group 1 comprised 50 pregnant women with uncomplicated, uneventful pregnancies without significant complications. In contrast, Group 2 encompassed 50 pregnant women presenting with clinical indicators of threatened miscarriage (pregnancies characterized by the presence of positive fetal heart sounds, concurrent vaginal bleeding, and a closed cervix).

### Inclusion criteria

Inclusion criteria for study participation encompassed pregnant women aged 20 to 35 years, exhibiting gestational ages between 7–13 weeks, and confirming the presence of a singleton embryo with detectable cardiac activity via ultrasound. Additionally, gestational age determined by the last menstrual period (LMP) was required to align within a 3-day window of ultrasound-derived crown-rump length.

### Exclusion criteria

Multiple pregnancies, previous hormonal treatment or progesterone supplementation, pre-existing medical conditions such as diabetes, thyroid disease, or antiphospholipid syndrome, as well as specific gynecological conditions including fibroids/adnexal masses, uterine malformations, obesity, ectopic pregnancy, and molar pregnancy, were grounds for exclusion from the study.

### Study procedures

Participants underwent a comprehensive clinical assessment encompassing a detailed medical history and physical examination, including abdominal, pelvic, and bimanual examinations. Furthermore, a battery of laboratory investigations was performed, including assessments of complete blood count, renal function (serum creatinine, serum urea), liver function (ALT, AST), and coagulation profile (Prothrombin time, prothrombin concentrations, INR). Additional laboratory parameters included quantitative hCG, progesterone levels, high-sensitivity CRP, and RH/ABO blood typing. Transvaginal ultrasound examinations were conducted to verify fetal viability, exclude structural anomalies, and establish precise gestational age.

### Outcome measures

The primary outcome measure in this study was the determination of serum C-reactive protein (CRP) levels, which were meticulously compared between the cohort presenting with threatened miscarriage and the control group. Serum CRP assessments were performed utilizing blood samples collected at the study’s commencement (between 6 – 14 weeks gestation) to elucidate potential discrepancies between groups and investigate the correlation of CRP levels with the risk of threatened miscarriage during early pregnancy.

### Secondary outcome measures

Secondary outcome measures comprised the assessment of ongoing pregnancy rates at 24 weeks gestation and miscarriage rates before the 20-week gestational mark. These outcome measures were systematically compared between the group of women presenting with threatened miscarriage and the control group characterized by uncomplicated pregnancies. The objective was to discern whether notable disparities in outcomes existed between these two distinct cohorts.

## Ethical Considerations

### Ethical approval

The study protocol underwent a rigorous ethical review process and received approval from the Institutional Review Board at Al-Azhar University and the Ethical Committee of Al-Azhar Faculty of Medicine. This approval underscores the study’s adherence to established ethical standards and principles.

### Informed consent

Before participating in the study, all enrolled patients provided written informed consent in a face-to-face interaction. This consent process included a comprehensive explanation of the research objectives and the various study procedures. Ensuring patient autonomy and comprehension was paramount throughout the informed consent process.

### Confidentiality and privacy

The study meticulously upheld the principles of patient confidentiality and privacy at every stage of the research endeavor. Stringent measures were in place to safeguard the sensitive personal information of study participants.

## Data Management and Analysis

### Data collection

Information gleaned from medical histories, clinical examinations, laboratory investigations, and outcome measures underwent a systematic coding process. This coded data was subsequently entered into Microsoft Excel for organized management.

### Statistical analysis

The data compiled within Microsoft Excel was subsequently imported into the Statistical Package for the Social Sciences (SPSS version 20.0) for comprehensive analysis. Qualitative data was presented as counts and percentages, whereas quantitative continuous data was expressed as mean values accompanied by their respective standard deviations. Various statistical tests, including Pearson’s correlation, Spearman’s correlation, and other appropriate tests, were meticulously employed to discern differences between groups and assess statistical significance. For statistical significance, a P value threshold of < 0.05 was considered statistically significant, while a P value of < 0.001 was deemed highly significant.

## Results

In this comprehensive study investigating potential predictors of first-trimester miscarriage, our analyses revealed several significant findings across demographic, laboratory, and ultrasound parameters. Independent t-tests showed no statistically significant differences between the patient and control groups for age (p = 0.539) or BMI (p = 0.338). However, ultrasound parameters exhibited substantial variations. Notably, gestational sac diameter (GSD) was significantly different between groups (p < 0.001), emphasizing its potential utility as a predictor.

Table 1 shows no statistically significant difference between groups regarding age and BMI.

Table 2 shows that progesterone was significantly lower among the patients’ group than in the control group. However, there is no significant difference between the groups regarding HCG.

Table 3 showed that CRP was significantly higher among the patients group than the control group. However, there is no significant difference between the groups regarding PAPP-A.

Table 4 shows a significant difference between the groups regarding the ultrasound parameters.

Table 5 shows a significant negative correlation between hs-CRP and FHR among the patients group.

Table 6 shows that hs-CRP was significantly higher among miscarriage patients compared to patients who continued pregnancy in the patients’ group. However, hs-CRP was higher among miscarriage patients compared to patients who continued pregnancy in the control group but without a statistically significant difference.

Specific laboratory parameters were scrutinized for associations with threatened miscarriage.

High-sensitivity C-reactive protein (hs-CRP) levels were significantly higher in patients versus controls (p < 0.001), suggesting an association between inflammation and miscarriage risk. Patients who miscarried had markedly higher hs-CRP levels compared to those with ongoing pregnancies (p < 0.001).

Additionally, correlation analysis revealed a significant negative association between hs-CRP and fetal heart rate (FHR) in patients (r = – 0.331, p = 0.019), indicating higher hs-CRP related to lower FHR. Receiver operating characteristic curves demonstrated hs-CRP’s predictive potential, with a cutoff > 9.3 mg/L showing 65.2% sensitivity and 89.6% specificity for first-trimester miscarriage prediction.

In summary, these robust findings provide nuanced insight into relationships between demographic, laboratory, and ultrasound parameters in the context of first-trimester miscarriage prediction.

## Discussion

Threatened miscarriage is characterized by the identification of a fetus exhibiting positive cardiac activity within a gestational age of less than 20 weeks, accompanied by vaginal bleeding and a closed cervix.^9^ It represents the most common complication of early pregnancy, with an incidence ranging from 14% to 20%.^12^ Beyond six weeks gestation, threatened miscarriage carries an approximate 10% risk of progressing to complete miscarriage. Additionally, it has been associated with adverse long-term pregnancy outcomes, including preterm delivery, placental abruption, intrauterine growth restriction, and low birth weight.^13^

Our study observed no statistically significant differences in age and BMI between the two groups. These findings are consistent with those reported by Jauniaux et al., who conducted a study assessing the role of hs-CRP in predicting and managing miscarriage in women with

first-trimester bleeding.^14^ Their study, including 71 threatened miscarriage cases (Group A) and 71 asymptomatic controls (Group B), matched for gestational age, maternal age, BMI, and smoking status, found no significant differences between groups for age and BMI.

The study found no significant differences between the two groups concerning routine laboratory parameters, including hemoglobin, total leukocyte count, platelets, alanine aminotransferase, aspartate aminotransferase, urea, and creatinine. However, it’s noteworthy that other studies have reported conflicting findings. Abdelsamie et al. discovered significantly higher mean values for white blood cells, neutrophils, monocytes, and lymphocytes in the study group than controls.^15^ In a related context, Cohen et al. aimed to investigate C-reactive protein (CRP) levels in early pregnancy to detect abnormalities, particularly ectopic pregnancy.^16^ They found significantly higher CRP levels in normal pregnancy (Group A) versus abnormal pregnancies (Group B + C and Group B alone), despite no differences in age and BMI between groups.

Our study revealed significantly higher CRP levels in the patient group than in controls, though no significant difference in PAPP-A was observed between groups. These results align with Cohen et al., who reported significant differences between their study groups for CRP levels.^16^ Nikbakht et al. also found a mean serum CRP level of 4.6 ± 2.7 mg/L in patients, along with 89.16% normal fetuses, 10% preterm births, and 5% small-for-gestational-age births.^17^

A notably higher miscarriage rate was observed in the patient group compared to controls. This finding is consistent with Jauniaux et al., who found no significant difference in miscarriage rates between their study groups.^14^

Our study established a significant negative correlation between hs-CRP and fetal heart rate in the patient group. Similarly, Bondarenko et al.

found that increased hs-CRP levels were associated with fetal disorders, underscoring its role as an inflammation marker in complicated pregnancies.^18^

We found that hs-CRP was significantly higher in miscarriage patients compared to ongoing pregnancy patients within the patient group but not in controls. These findings agree with Jauniaux et al., who reported significant differences between their study groups for hs-CRP levels.^14^ Abdelsamie et al. assessed maternal serum hs-CRP and differential leukocyte count (DLC) for diagnosing threatened miscarriage in 100 pregnant women, concluding a significant difference between study groups for hs-CRP levels.^15^

In our study, only gestational sac diameter (GSD) significantly predicted first-trimester miscarriage, with 71.4% sensitivity and 70.9% specificity. Tadmor et al. similarly found that the GSD and crown-rump length ratio predicted miscarriage in a prospective cohort, demonstrating a sensitivity of 78.3% and specificity of 97.8%.^19^

## Conclusion

In conclusion, our study provides evidence that pregnant women with threatened miscarriage exhibit significantly elevated serum hs-CRP levels compared to controls, suggesting the potential utility of hs-CRP as a predictive marker in such cases. These findings contribute to the evolving understanding of the multifaceted factors associated with threatened miscarriage and underscore the importance of exploring serologic markers in conjunction with established sonographic parameters for enhanced prognostication.

## Declarations

### Ethics approval and consent to participate

The study received ethical approval from the university’s faculty of medicine’s ethics committee, and all enrolled participants provided written consent to participate in the research.

## Consent for Publication

Written consent was obtained from all participants, granting permission to use their data in this publication.

## Availability of Data and Materials

The datasets utilized and/or analyzed during this study are accessible from the corresponding author upon reasonable request.

## Competing Interests

The authors assert they have no competing interests to disclose.

## Funding

This research received no specific grant from any funding agency, whether in the public, commercial, or not-for-profit sectors.

## Authors’ Contributions

All authors of this manuscript made equal and substantial contributions to the study. They have collectively reviewed and approved the final version of the manuscript.

## Acknowledgments

We express our sincere gratitude to the attending physicians, esteemed consultants, dedicated colleagues, invaluable mentors, and the patients, who actively participated in and contributed significantly to the success of this research endeavor.

## Figures and Tables

**Figure 1.**
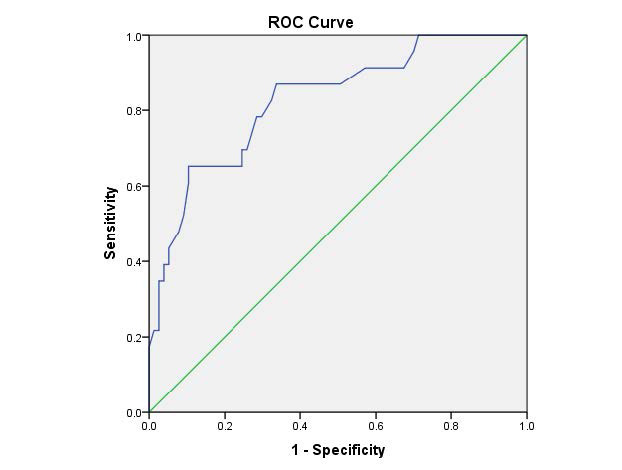
ROC curve for hs-CRP as a predictor for miscarriage in the first trimester.

**Figure 2.**
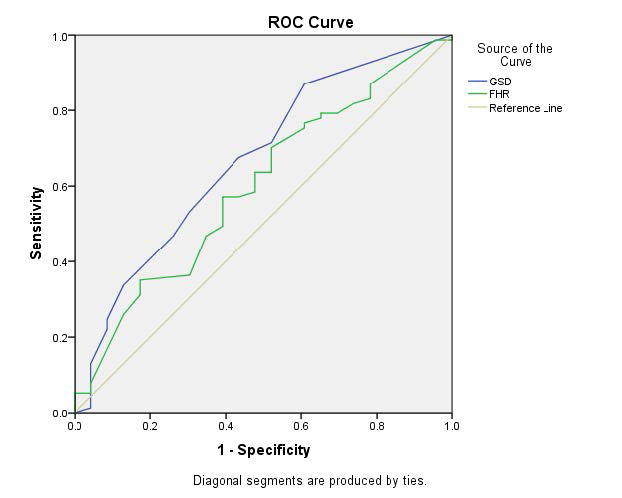
ROC curve for possible US parameters as a predictor for miscarriage in the first trimester.

**Table 1.** Demographic distribution between the two groups.

Variables	Patients (n = 50)	Controls (n = 50)	t	P
**Age** (years)	27.1 ± 3.47	26.64 ± 3.98	0.616	0.539
Mean ± SD				
**BMI** (kg/m^2^)	27.29 ± 2.18	26.88 ± 2.08	0.962	0.338
Mean ± SD				

BMI: body mass index; t: Independent T-test.

* P-value ≤ 0.05 indicates significant, p < 0.001 indicates highly significant, P > 0.05 indicates non-significant.

**Table 2.** Gonadotropic laboratory parameters between the two groups.

Variables	Patients (n = 50)	Controls (n = 50)	t	P
**HCG** (mIU/ml)	58458	71227	**MW 242**	**0.569**
Median (range)	(1300 – 280665)	(2500 – 289540)		
**Progesterone (ng/ml)**	18.95 ± 6.14	24.63 ± 6.67	**4.43**	**< 0.001**
Mean ± SD				

HCG: Human chorionic gonadotropin; t: Independent T-test; MW: Mann-Whitney test.

* P-value ≤ 0.05 indicates significant, p < 0.001 indicates highly significant, P > 0.05 indicates non-significant.

**Table 3.** Specific laboratory parameters between the two groups.

Variables	Patients (n = 50)	Controls (n = 50)	MW	P
**PAPP-A** (mU/mL)	0.083 ± 0.041	0.084 ± 0.034	1141	0.448
Mean ± SD				
**hs-CRP** (mg/L)	7.86 ± 3.27	5.63 ± 2.56	**730**	**<** 0.001
Mean ± SD (Range)	(3.2 – 18.2)	(1 – 12.5)		

PAPP-A: pregnancy-associated plasma protein-A; hs-CRP: high-sensitivity C-reactive protein; MW: Mann-Whitney test.

* P-value ≤ 0.05 indicates significant, p < 0.001 indicates highly significant, P > 0.05 indicates non-significant.

**Table 4.** Ultrasound parameters between the two groups.

Variables	Patients (n = 50)	Controls (n = 50)	t	P
**CRL** (mm)	9.03 ± 2.88	11.61 ± 4.46	**MW 792**	**0.002**
Mean ± SD				
**GSD** (mm)	20.56 ± 1.96	25.18 ± 2.42	**11**	**< 0.001**
Mean ± SD				
**GSV (mm^3^)**	3.32 ± 0.611	3.63 ± 0.531	**2.71**	**0.008**
Mean ± SD				
**FHR** (beat/min)	131.1 ± 23.75	150.9 ± 16.49	**4.84**	**< 0.001**
Mean ± SD				

CRL: crown-rump length; GSD: gestation sac diameter; GSV: gestation sac volume; FHR: Fetal heart rate; t: Independent T-test; MW: Mann-Whitney test.

* P-value ≤ 0.05 indicates significant, p < 0.001 indicates highly significant.

**Table 5.** Correlation between CRP and other parameters among studied groups.

hs-CRP	Patients		Controls	
	R	P	R	P
**HCG**	0.085	0.556	0.238	0.097
**Progesterone**	0.141	0.327	0.209	0.144
**PAPP-A**	0.084	0.562	0.024	0.867
**CRL**	–0.180	0.212	–0.063	0.664
**GSD**	–0.054	0.711	–0.085	0.559
**GSV**	0.102	0.480	0.024	0.867
**FHR**	**–0.331**	**0.019**	–0.098	0.500

HCG: Human chorionic gonadotropin; hs-CRP: high-sensitivity C-reactive protein; PAPP-A: pregnancy-associated plasma protein-A; CRL: crown-rump length;

GSD: gestation sac diameter; GSV: gestation sac volume; FHR: Fetal heart rate; r: correlation coefficient.

* P-value ≤ 0.05 indicates significant correlation, P > 0.05 indicates non-significant correlation.

**Table 6.** hs-CRP levels among the studied groups according to outcome.

Patients group	Miscarriage (n = 16)	Continue (n = 34)	MW	P
**hs-CRP** (mg/L)	10.99 ± 3.03	6.38 ± 2.17	**54**	**< 0.001**
Mean ± SD			
**Control group**	**Miscarriage (n = 7)**	**Continue (n = 43)**	**MW**	**P**
**hs-CRP** (mg/L)	6.7 ± 2.36	5.46 ± 2.58	**99**	**0.157**
Mean ± SD			

hs-CRP: high-sensitivity C-reactive protein; MW: Mann-Whitney test.

* P-value ≤ 0.05 indicates significant, p < 0.001 indicates highly significant, P > 0.05 indicates non-significant.

**Table 7.** The cutoff statistics for hs-CRP in predicting miscarriage in the first trimester.

Cutoff	AUC	S.E.	Sig.	95% Confidence Interval	Sensitivity	Specificity
**9.3 mg/L**	0.826	0.050	**< 0.001**	0.729 – 0.924	65.2%	89.6%

hs-CRP achieved a significant result in predicting miscarriage in the first trimester at cutoff > 9.3 mg/L with a sensitivity of 65.2% and specificity of 89.6% (Table 7 and Figure 1).

**Table 8.** The statistics for GSD and FHR in predicting miscarriage in the first trimester.

Variable	AUC	S.E.	Sig.	95% Confidence Interval	Sensitivity	Specificity
**GSD**	0.669	0.065	**0.014**	0.541 – 0.797	71.4%	70.9%
**FHR**	0.599	0.067	0.151	0.468 – 0.730	70.1%	69.6%

Only GSD achieved significance in predicting miscarriage in the first trimester, with a sensitivity of 71.4% and a specificity of 70.9% (Table 8 and Figure 2).
